# The perceptions of palliative care medical practitioners towards oral health: A descriptive qualitative study

**DOI:** 10.1177/02692163241233974

**Published:** 2024-03-20

**Authors:** Amy R. Villarosa, Meera Agar, Ariana Kong, Mariana S Sousa, Janeane Harlum, Deborah Parker, Ravi Srinivas, Jennifer Wiltshire, Ajesh George

**Affiliations:** 1 Australian Centre for Integration of Oral Health (ACIOH), School of Nursing and Midwifery, Western Sydney University, Penrith, NSW, Australia; 2 Ingham Institute for Applied Medical Research, Liverpool, NSW, Australia; 3 National Centre of Epidemiology and Population Health, Australian National University, Canberra, ACT, Australia; 4 IMPACCT (Improving Palliative, Aged and Chronic Care through Clinical Research and Translation), Faculty of Health, University of Technology Sydney, Sydney, NSW, Australia; 5 Department of Palliative Care, South Western Sydney Local Health District, Liverpool, NSW, Australia; 6 District Palliative Care Service, South Western Sydney Local Health District, Liverpool, NSW, Australia; 7 Oral Health Services, South Western Sydney Local Health District, Liverpool, NSW, Australia; 8 School of Dentistry, Faculty of Medicine and Health, University of Sydney, Camperdown, NSW, Australia; 9 School of Nursing, Faculty of Science, Medicine and Health, University of Wollongong, Wollongong, NSW, Australia

**Keywords:** Palliative care, oral health, patient care health, referrals, focus groups, medical practitioners

## Abstract

**Background:**

Oral health problems are common, but often overlooked, among people receiving palliative care.

**Aim:**

To better understand how oral health can be addressed in this population, this study aimed to explore the perceptions of oral health care among medical practitioners who provide palliative care to inform the development of a palliative oral health care program.

**Design:**

A descriptive qualitative design was adopted.

**Setting/participants:**

A single focus group was conducted with 18 medical practitioners at a palliative care facility in Sydney, Australia. All participants had experience providing palliative care services to clients. The focus group was audio recorded, transcribed and thematically analysed.

**Results:**

The results from the inductive thematic analysis identified four themes. The themes highlighted that participants were aware of the oral health needs of people receiving palliative care; however, they also reflected on the complexity in delivering oral health care across the healthcare settings, as well as the challenges around cost, lack of appropriate dental referral pathways, time constraints and limited awareness. Participants also provided recommendations to improve the delivery of oral health care to individuals receiving palliative care.

**Conclusions:**

To improve the provision of oral health care in this population, this study highlighted the need for oral health training across the multidisciplinary team, standardised screening assessments and referrals, a collective responsibility across the board and exploring the potential for teledentistry to support oral health care provision.

**What is already known about the topic?**

- Poor oral health among people receiving palliative care is common and can impact on wellbeing and quality of life.
- In Australia, palliative care is often coordinated by a medical practitioner.
- Medical practitioners could play a role in integrating oral health care into the management plan of people receiving palliative care services.

**What this paper adds?**

- Medical practitioners are aware about the oral health needs of clients.
- Numerous challenges affect the provision of oral health care in palliative and non-palliative care settings.
- Improving the provision of oral health care requires additional training, coordination of the multidisciplinary team and accessible dental referral pathways.

**Implications for practice, theory or policy**

- An integrated approach of the multidisciplinary team, including carers, in the palliative care setting could enhance the provision of oral health care.
- Further oral health education and training is necessary to improve the capacity of palliative care providers to provide integrated care.
- Remote health services, like teledentistry, could facilitate care and expedite dental referrals.

## Introduction

The needs of people who require palliative care are diverse, and will depend on the progression of their illness.^1^ As the illness progresses, individuals may require support from a multidisciplinary team to assist with daily activities.^2^ A significant care requirement for people receiving palliative care is oral health.^2,3^ Depending on the progression of the illness, people will have increasing oral health needs yet may not be able to sustain oral health self-care.^2,3^

Including oral health as part of palliative care can often be overlooked by palliative health care providers.^4,5^ People receiving palliative care will most commonly experience xerostomia, oral candidiasis, dysphagia, orofacial pain, alterations to taste and oral ulcerations.^3,6^ These oral health problems can significantly impact on a person’s ability to eat, swallow and speak with others.^6^ Consequently, some people may become more anxious, depressed or ashamed about their oral health.^6,7^ To effectively address oral health among people receiving palliative care, oral health care needs to be managed as part of a person’s care plan.

Internationally, palliative care may be managed by a medical practitioner, advanced nurse specialists or by a palliative care team.^8–11^ In Australia, palliative care is delivered through numerous health care providers that are largely coordinated by a medical practitioner.^12,13^ Depending on the nature of the illness and accessibility to palliative care services, this may involve palliative care specialists or a primary care provider like a general practitioner.^13^ Medical practitioners often coordinate ongoing care and assist in managing physical symptoms, for example by prescribing the appropriate medications, or by referring a person to other health care providers.^14,15^ Medical practitioners could improve oral health by prescribing appropriate medications to treat certain oral conditions or outline an oral care regimen. Yet little is known regarding the perceptions of medical practitioners regarding the provision of oral care to people receiving palliative care. Understanding the perspectives of medical practitioners is important to develop and integrate a model of care to improve the oral health of people receiving palliative care. This study aimed to explore the perceptions of oral health care among medical practitioners who provide palliative care, as well as their perceptions regarding the implementation of an oral health care program for people receiving palliative care.

## Materials and methods

### Design

A descriptive qualitative design was adopted. This study is part of a larger project to develop a palliative oral health (PALLIOH) model within the Greater Sydney region to improve the quality of oral health care for people with life-limiting illnesses. The perceptions of palliative care nurses were also explored in another study.^16^

### Context

This study was conducted at a local health district in Greater Western Sydney between October 2017 and August 2018. This district had a population of approximately 1 million people, under half (46%) of whom spoke English at home, and who experienced anywhere from the first to the tenth decile of the Index of Relative Socio-economic Disadvantage (IRSD).^17,18^

### Eligibility criteria

This study aimed to recruit medical practitioners working in south western Sydney. A medical practitioner in Australia is a qualified doctor who is responsible for diagnosing and managing conditions; provides recommendations for preventative care; and refers clients to specialists or other health care services.^19^ This study included any person with an Australian-recognised medical practitioner qualification, including those who were not palliative care specialists. Although the study targeted medical practitioners working in palliative care services, no exclusion criteria were defined.

### Sampling

A purposive sampling technique was employed to recruit medical practitioners working in palliative care services in the southwestern Sydney area.

### Recruitment

Flyers and information sheets were distributed through email invitations to all eligible medical practitioners working in palliative care services across the district. Word-of-mouth was also used. People who were interested were invited to contact the researchers for further information. The option to participate in telephone interview was also available; however, all participants were able to attend the focus group.

### Ethical considerations

Ethical approval was received from the South Western Sydney Local Health District Human Research Ethics Committee (HE17/007). All participants received verbal and written information about the study and provided written consent. The focus group was conducted in-service. There were no previous relationships between any of the participants and study investigators who collected or analysed the data.

### Data collection

After collecting demographic information, a focus group was facilitated by two experienced researchers – AG (male, PhD) and ARV (female, MBiostat) using a topic guide (Supplemental Material). The interview guide was developed and refined by the consultation group, who had expertise in research, public oral health and palliative care. The focus group aimed to explore the experiences of medical practitioners in promoting oral health, as well as some of the challenges they faced and potential solutions to mitigate some of the concerns that were discussed. The facilitators directed the focus group to ensure that all participants had an opportunity to share their opinions and experiences, and asked probing questions where appropriate for additional relevant information. The focus group was conducted in a private room at a palliative care facility, was audio-recorded and lasted 49 min.

### Data analysis

The recording was sent to a professional transcription service. Braun and Clarke’s framework for inductive thematic analysis was used to guide analysis so that the varied perspectives shared among participants in the focus group could be captured.^20^ To capture the breadth of perspectives, the team had a consensus that the analysis should produce a rich description of the data set and that the analysis should take place at a semantic (explicit) level.^20^

As part of the first phase, *Familiarisation*, both ACK and ARV read the transcripts for immersion, noting initial ideas. At the same time, ARV checked the transcript for accuracy and uploaded the transcript onto NVivo. For the next phase, *Generating initial codes*, ARV inductively coded the transcript independently. In the third phase, *Search for themes*, these codes were independently sorted into categories by ACK and ARV, who kept memos during the analysis, until preliminary themes were developed. As part of *Reviewing themes* and *Defining and naming themes* in the fourth and fifth phases, both ACK and ARV had a consensus meeting with AG to create a list of themes and subthemes. These were shared with the rest of the team for clarification, discussion and to finalise the list of themes and sub-themes. For the final phase, *Producing the report*, ACK and ARV identified relevant quotes for each theme from NVivo to illustrate the concept. All identifying data was removed to ensure confidentiality, and coded pseudonyms were allocated. Pseudonym codes followed the pattern MF01, where the first letter represented medical practitioner, the second letter represented sex (M = male and F = female) and the two-digit number represented the order of participants’ first appearance in the transcript.

### Enhancing trustworthiness

A process of peer-coding and peer debriefing were integrated into the analysis to enhance trustworthiness. The two researchers (ACK, ARV) who independently collapsed the codes to create categories and themes discussed differences in thematic structures with a third researcher (AG) until a consensus was reached. Investigators who collected and analysed the data debriefed with the larger team throughout the study to discuss the methodological decisions and findings to increase credibility.

### Reflexivity

ARV and ACK are researchers with experience in qualitative and public oral health research. AG is a trained dentist with expertise in public oral health research. MA, DP, LW, JH and RS have clinical expertise, health service and research experience in either palliative care or oral health care.

## Results

### Participant characteristics

The single focus group was attended by 18 medical practitioners. The mean age of participants was 41.17 (±5.628) with a range 36–57 years old. Two thirds of participants were female and had on average 6 (range 0–20) years of experience working in palliative care settings. Over half (61%) were staff specialists or senior staff specialists, one third were registrars (33%) and one participant was a general practitioner (GP).

### Thematic analysis

A total of 271 initial codes were generated. Following analysis, four distinct main themes were constructed (see Table 1).

**Table 1.** Themes and sub-themes from analysis.

Theme	Subthemes
Good oral health is important in palliative care management	Unique (yet ubiquitous) oral health needs
	Oral health a matter of dignity and quality of life
‘Rings of care’: From the hospital to the community	Oral care being a shared role across rings of care
	Inconsistent levels of oral care between settings
How the current model restricts the appropriate oral health provision	Cost of oral health care
	Lack of appropriate dental referral pathways
	Time constraints and prioritisation within the healthcare system
	Limited awareness and adherence
	Lack of training and resources
Strategies to improve oral health care provision for people receiving palliative care	Multidisciplinary oral health training
	Standardised oral health screening
	Clear and accessible referral pathways
	Collective responsibility across the rings of care
	Connecting the rings of care through teledentistry

### Theme 1: Good oral health is important in palliative care management

Participants were cognisant of the importance of oral care in their palliative care management plan. They discussed how the oral health care needs of people receiving palliative care were unique, and yet ubiquitous among their clients. Medical practitioners also connected the importance of maintaining good oral health to clients’ sense of dignity, physical wellbeing and quality of life.

#### Unique (yet ubiquitous) oral health needs

Participants acknowledged that palliative care introduced oral health problems, including denture problems because of xerostomia, or for clients with motor neurone disease, sialorrhea. Participants discussed that the severity of oral health issues experienced varied according to diagnosis, with head and neck patients having the most significant dental problems because of the post-treatment effects. The observed frequency of oral health problems reported varied according to site.

*‘One of the. . . most common symptoms we see is dry mouth’ (MF03)*

*‘There are often lots of issues [relating to oral health]’ (MF01)*

The oral care needs of people receiving palliative care were unique and complex because of the need for medical practitioners to consider whether their clients could receive general anaesthesia, even if the oral problem presented significant problems to overall well-being. Some medical practitioners also recounted treating symptomatic dental problems while their clients were waiting for a dental appointment.

*‘Are they well enough to have a general anaesthetic to have a tooth removed, it’s that catch-22’ (MF01)*

*‘[we need to treat] some dental abscesses whilst waiting for a dental appointment’ (MF01)*

#### Oral health a matter of dignity and quality of life

Most participants agreed that oral health care was important for people receiving palliative care because of its multifaceted impact on clients’ wellbeing and quality of life. Participants discussed how poor oral health can affect general health outcomes, causing difficulties with pain management which can lead to more debilitation and subsequently poorer mortality outcomes. They described the impact of oral health on clients’ nutritional intake, ultimately resulting in patients presenting with weight loss. Medical practitioners acknowledged that the impact was not just physical; some linked the impact of oral health problems on a person’s mental and social wellbeing, including their speech and self-image.

*‘if they’re not getting mouth care, they just get this glued, crispy interior’ (MF08)*

*‘More debilitation, which can then lead to poorer outcomes generally in terms of mortality, never mind the mobility aspect’ (MM07)*

### Theme 2: ‘Rings of care’: From the hospital to the community

**Figure 1.**
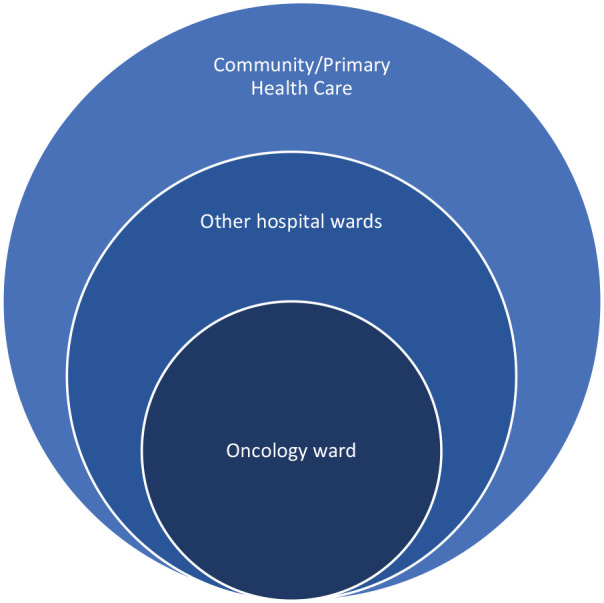
Rings of care for people receiving palliative care, as described by a participant.

One participant described the care given to people receiving palliative care as ‘*rings of care*’ (Figure 1). The oncology ward was recognised as being at centre of these rings, yet not all clients had an oncological problem or needed to present to the oncology ward. In the other rings, oral care provision appeared to become less of a priority:

*‘To paint a picture of [the] consult service, you have rings of care and interest, so the oncology ward, I can have quite a big impact . . . this pathway is important and these are the outcomes. . .This is my management plan. . . But the further away I get from the [oncology] ward towards surgical, gastroenterology, the less of an interest there is and the less follow up.’ (MF09)*

#### Oral care being a shared role across rings of care

Participants discussed the roles that staff specialists, ward nurses, GPs, community nurses and unpaid caregivers play in providing oral care. It appeared that there were no strict boundaries with each person’s role, which was dependent on the client’s prognosis which guided how extensively they were going to be involved. Medical practitioners listed their responsibilities in oral care to include identifying issues, writing regular mouth care on the medication chart and referring clients to a specialty service for more complication needs. Participants also recognised their role providing treatment and education where possible on the ward.

*‘Does depend on what the prognosis is as how extensive you’re going to be involved’ (MF02)*

*‘If I see an ulcer . . . I’ll take a viral swab, so if it is something that I can treat, at least I can do something to treat the cause of the ulcer’ (MF06).*

*‘Reinforcing the oral hygiene that we do’ (MF01).*

There was a consensus that nurses play a role in oral care provision because of their contact with the client and because of their expertise on proper mouth care. There was also agreement that nurses played a role in oral health assessment both on the ward and in the community.

*‘We’re leaving a lot of it to them [nurses] because they’re with the patient all the time’ (MF06)*

*‘I tend to rely on [nurses’] advice quite a lot . . . technique of how to do proper mouth care’ (MM10) ‘Oral health is included in every report that they [nurses] write’ (MF02)*

Within the community, GPs were often required to play a role in maintaining oral health among people receiving palliative care. One participant recounted that people receiving palliative care see GPs for dental problems due problems with timely dental access. Participants perceived the role of unpaid caregivers, including family members, was to assist with daily oral care for people receiving palliative care. There was agreement that this was also beneficial for carers as they could have agency in improving the recipient’s quality of life.

*‘They’re coming to see you because they need help and they haven’t been able to access a dentist’ (MM07)*

*‘it [assisting with oral care] empowers carers, especially towards end of life,’ (MM10)*

#### Inconsistent levels of oral care between settings

The participants reflected on inconsistencies in attitudes towards prioritising oral health care in palliative care settings, and discussed the need for oral health screening or more structured processes in non-palliative care settings. Even when medical practitioners prescribed oral care regimens for clients moved to other settings, they observed poor compliance in these settings.

*‘[I] make a concerted effort for every one of my patients at [site name] to make sure – have a look at their mouth et cetera because I know as soon as we leave as a [palliative care] consult service it won’t get done.’ (MM10).*

*‘Outside of palliative care unit it’s much more ad hoc. Normally they’re often just left for the patient to do’ (MF05).*

*‘. . . even with prescribing . . . the mouth care, you often go back, and it’s written S-S-S-S-S, saying the patient has done it themselves. You go see the patient. They cannot possibly [do it themselves]’ (MF02).*

Some acknowledged that there were inconsistencies in performing and documenting oral health care and oral health assessments across palliative care settings.

*‘I don’t think it’s [oral care] always routine’ (MF06)*

*‘nurses have a form, but doctors don’t, and I think it’s [oral care] poorly recognised,’ (MM10)*

*‘I think sometimes it’s [oral care] neglected because it’s not so obvious. . . I haven’t always done the best I could’ (MF05).*

### Theme 3: How the current model restricts the appropriate oral health provision

There was considerable discussion surrounding the challenges to the provision of oral care. Participants identified how the existing model, with respect to the healthcare system structure and their current training, limited clients from receiving timely and appropriate dental treatment. These challenges included cost, lack of dental referral pathways, time constraints and prioritisation, limited awareness and adherence and lack of training and resources.

#### Cost of oral health care

Participants commented that when it came to clients and their ability to receive oral treatment, it was sometimes a financial decision. The cost of dental care was seen as preclusive, which contributed to some individuals seeing their GP instead of a dentist. Even obtaining personal oral care supplies were perceived as costly. Although medical practitioners highlighted that providing ergonomic toothbrushes could increase the ease of oral care, it may not be affordable for some people receiving palliative care.

*‘a lot of patients in outpatients with bad teeth, but they can’t afford to go to the dentist’ (MF05).*

*‘because they get a Medicare rebate [at a GP] . . . but they don’t get that from the dentist’ (MM07).*

#### Lack of appropriate dental referral pathways

Participants recognised the need for appropriate and timely dental referral pathways for those requiring dental treatment; thus, it was seen as difficult to access dental care for individuals receiving palliative care, particularly for inpatients.

*‘When you’re in hospital with an inpatient who’s not looking like they’re going to get home any time soon and thinking about having to take them to an external service [like dentist], that seems like a path of far too much resistance for everybody.’ (MF06)*

Participants’ knowledge and access to available public dental services were also inconsistent between sites. While some participants were not aware of the dental referral pathways available, others reflected that they utilised referral pathways intended for other districts due to their accessibility.

*‘At [site name], I've got to say, we can usually refer up to [site name] Dental Clinic. They’re usually fairly responsive’ (MF01)*

*‘Here, to be honest, I don’t know who to call. I’d probably have to do some [routine] general phone calls to find someone who’s willing to listen’ (MF09).*

Even when dental care could be accessed for clients, medical practitioners considered that could be difficult to cater for their mobility needs. Some also perceived that some clients may refuse treatment because of the client’s belief that dental treatment would be ineffective.

*‘those that are non-mobile, bedbound. . . Some of them are just never going to get into a dental chair’ (MF02)*

*‘[the belief that] what’s going on in their mouth is not going to get any better’ (MF01).*

#### Time constraints and prioritisation within the healthcare system

One participant highlighted that palliative care providers, including nurses, could experience the challenge of time constraints. With a finite amount of time, there are competing priorities so they also admitted that they may need to rush through oral assessments.

*‘I take a cursory look, open your mouth. . .It might be a 10, 15 second assessment’ (MF06).*

#### Limited awareness and adherence

The lack of oral health awareness and adherence to oral care regimens of people receiving palliative care were also discussed. Specifically, participants identified the need for individuals to understand how oral health could impact on their quality of life. At the same time, they recognised that adherence to oral care regimens was influenced by other factors such as the individual being able to remember to perform oral care, hand dexterity or nausea and pain associated with a medical condition.

*‘There might be an under-recognition of the fact that it [poor oral health] actually impacts on their symptoms and their quality of life’ (MF06)*

*‘Some of it’s compliance. Some of it’s just do they actually do it [oral care], do they remember to do it, the taste of various things?. . . Their dexterity to open a container. . .’ (MF01)*

*‘I often say do it [oral care] after each meal and before bed, but if they’ve had a struggle with the meal because they’re nauseated or they’ve got pain in their mouth or something, then they’re not always going to do what we advise.’ (MF01)*

#### Lack of training and supplies

Participants highlighted the lack of formal oral health training and instead relied on asking or observing colleagues, or on conjecture. Furthermore, medical practitioners commented that hospital oral care supplies were limited or did not stock ideal oral care products.

*‘We don’t get any formal training [in oral health], so it’s all Chinese whispers. . .’ (MF04)*

*‘I never really know what’s the appropriate swab to use’ (MF08)*

*‘On certain wards they’ve gotten rid of all of the swabs and got these little spongy toothbrush things, which actually are very sharp’ (MF09)*

### Theme 4: Strategies to improve oral health care provision for people receiving palliative care

There was in-depth discussion regarding strategies that could improve oral health care provision. These included the provision of multidisciplinary oral health training, standardised oral health screening, accessible referral pathways, collective responsibility across the rings of care and connecting the rings of care through teledentistry.

#### Multidisciplinary oral health training

Participants acknowledged the need for multidisciplinary oral health training across the palliative care team. Such training would need to be tailored to meet the responsibilities of each person in the team; for example, nurses on the general ward would need training to provide oral health care education, whereas medical oncologists would need to have education due to the medications they provide that affect oral health. Unpaid caregivers would also need training on how to provide oral care.

*‘educating the nurses on the ward, in a general ward’ (MM10)*

*‘the medical oncologist as well because they prescribe an awful lot of medications that affect the oral health’ (MF03)*

*‘[unpaid caregivers need] knowledge, how to do it’ (MF01)*

Participants shared their preferred modes of delivery for training, which varied but included a one-off training session that included hands-on simulations. There was a consensus that Continuing Professional Development points would incentivise this training.

*‘I’m happy to sit down and do an online thing. I’m also happy to go and do a half day or a full day’ (MF01)*

*‘I don’t think an online thing would be able to properly show you the intricacies of a mouth exam’ (MF04).*

Participants suggested this training could be used for both medical practitioners and other health professionals such as general ward nurses. For medical practitioners, the participants suggested topics that would inform their prescription for oral care regimens and referring clients to dental services, which included mouth case, tools or instruments, common symptoms, identifying dental emergencies and appropriate referral pathways.

*‘the most common things that we need to look out for [regrading dental problems]’ (MM10)*

*‘identifying dental emergencies’ (MF12)*

*‘how do you manage those different problems [dental] and what’s the referral pathway?’ (MM10)*

#### Standardised oral health screening

One participant discussed the need for a standardised oral health screening process to facilitate their oral health assessment, which would be clearly defined and easily administered to direct their next actions, particularly for high-risk patients.

*‘simple screening exam, so that you pick up the major problems. . .can do within a minute, that will really direct where I go from there. . . then you’d go a more detailed examination’ (MM10)*

#### Clear and accessible referral pathways

Participants also discussed the need for a clear, referral pathway that was easy-to-access to increase the number of their patients who received dental treatment. For private dental pathways, participants expressed the desire for a contact list for reliable and affordable dental care providers.

*‘A clear referral pathway which we know that is quite easy to access’ (MM10)*

*‘Different pathway for our patients - because they do have different needs’ (MF03)*

#### Collective responsibility across the rings of care

There was some discussion around the need for oral health to be a collective responsibility as it was an area of healthcare that is often managed separately. One participant expressed that oral care provision should be more integrated into other services, and another recalled a dentist at a site they used to work at where he would facilitate integrated care. Another participant highlighted the benefit of learning through interdisciplinary interaction with an occupational therapist, where they learned of an oral care instrument.

*‘It’s the head. It’s part of the body, why aren’t the dental services in the hospitals?’ (MF04)*

*‘{Dentist on site] who came up and saw consults. He would just look and make a spot diagnosis, and then he’d teach everyone who wanted to hear what it was.’ (MF06)*

*‘{some products]. . .which has got a toothbrush on each side, so actually really helps if you’re having difficulty and you’ve got manual dexterity problems. . . That’s where the occupational therapy side of things might come in, if you had good occupational therapist who would be aware of some of these products’ (MF05)*

#### Connecting the rings of care through teledentistry

There was general agreement that teledentistry could play a role to integrate the delivery of oral care to people receiving palliative care, particularly for clients with limited mobility or for people who needed an avenue to quickly access dental advice. Furthermore, participants saw that using teledentistry as a platform to improve communication between health professionals could improve information transfer when making referrals, and this would not be too different from what they already do. Others suggested a trained clinical nurse consultant (CNC) may be the best person to engage in teledentistry.

*‘We’ve got no options for community clients who can’t get out of the house’ (MF04)*

*‘For some of those patients that we can do a really simple fix because you [the dentist] know what you’re looking at, and we [medical practitioners] don’t’ (MF01).*

*‘I used to take photos of x-rays and send them to my orthopaedic friend and say where’s the fracture?’ (MF06)*

*‘the CNC might be the one person who could maintain continuity’ (MF04)*

Nevertheless, participants also expressed some concerns regarding the limitations of teledentistry.

*‘taking a picture of the oral cavity may be still restrictive.’ (MF12)*

*‘There’ll be a lot of logistics around who’s taking that camera and how do we know where it’s going and coming back and getting the wireless system.’ (MF06)*

## Discussion

### Main findings

Most medical practitioners recognised the importance of oral health care for people receiving palliative care. Practitioners discussed the implications of oral problems for reduced dietary intake, greater pain levels, poorer quality of life and in symptom management for people receiving palliative care. Nevertheless, effective oral health care provision was undermined by gaps in knowledge, clear and accessible referral pathways to professional dental services, standardised management plans and uncertainty around the roles and responsibilities of each healthcare provider within multidisciplinary teams. This echoes a recent rapid review,^3^ which found that a lack of knowledge, training and professional dental care and uncertainty regarding the roles of healthcare providers, were barriers to oral care provision for people in palliative care in the United States, Japan and the United Kingdom.^21^ Most medical practitioners reported inconsistencies in oral health assessment and management between palliative care and non-palliative care settings. Inconsistencies in quality of care have been documented for palliative care provision, which have recommended improvements in knowledge transfer between general and palliative settings.^22^

Despite the numerous challenges related to healthcare system structures and limited training and education, medical practitioners discussed several strategies that could inform a future palliative model of oral health care. These strategies included the need for multidisciplinary oral health training, standardised screening processes, accessible referral pathways and a collective responsibility across the various services. Similar strategies may be beneficial for oral care as this study identified that knowledge could be strengthened through tailored oral health education for medical practitioners and nurses working with palliative care populations. Although the effectiveness of such interventions has not yet been explored in the palliative care setting, research in North America, Europe and Australia have highlighted that training can improve knowledge and attitudes regarding provision of oral health care.^23,24^

### Study implications

An integrated approach to palliative oral health care provision could improve quality of care and bridge the gap.^22^ Approaches to integrated oral health care provision, specifically involving caregivers, were discussed to improve linkage to dental services. While caregivers undertake multiple care responsibilities, it was suggested that they should also be actively involved in providing day-to-day oral care. Evidence suggests that despite American caregivers viewing oral health as an important responsibility, it may receive a lower priority.^25^ Thus, to ensure caregivers can be involved in integrated oral care models, a deeper understanding of the barriers and facilitators to their provision of oral health care is needed.^25^ This research should be conducted in conjunction with comprehensive and integrated oral health education and training involving the palliative multidisciplinary team.

Implementing remote online communication platforms into practice, such as teledentistry, could facilitate oral care provision and expedite dental referrals. Although there is limited evidence regarding teledentistry in the palliative care setting, the use of telehealth interventions has become more popular, particularly since the COVID-19 pandemic, as it has the potential to overcome barriers relating to access and staffing.^26^ Although there may be some technical limitations,^27^ research has demonstrated that teledentistry is acceptable to consumers internationally, has a high accuracy for dental diagnoses and allows time-efficient screening and triage.^26–28^

### Strengths and limitations of the study

The study has some strengths and limitations that should be considered. First, there may be selection bias. All participants practised in a metropolitan area in Australia, so the perspectives may not represent practitioners working in regional and rural or remote areas, or in other countries. Although there was a high proportion of female participants, the Australian specialist palliative medicine physician workforce is almost two-thirds female.^12^ Due to the voluntary nature of participation, participants in this study may have a higher than usual interest in oral health care. Second, only one focus group was conducted due to scheduling and funding constraints, so it was difficult to determine whether sufficient data had been collected; however, as there was a large number of participants, a rich and diverse range of perspectives were shared.

## Conclusion

This study offers novel insights into the medical practitioners’ perceptions of oral care provision to people receiving palliative care. The results suggest that more emphasis should be placed on multidisciplinary oral health continuing education and training, oral health screening and clear referral pathways for people receiving palliative care. Future research should focus on the development and evaluation of palliative oral health training programs, potential for teledentistry, integrated models of oral health care, as well as the barriers and facilitators experienced by caregivers and people receiving palliative care.

## Supplemental Material

sj-pdf-1-pmj-10.1177_02692163241233974 – Supplemental material for The perceptions of palliative care medical practitioners towards oral health: A descriptive qualitative study Supplemental material, sj-pdf-1-pmj-10.1177_02692163241233974 for The perceptions of palliative care medical practitioners towards oral health: A descriptive qualitative study by Amy R. Villarosa, Meera Agar, Ariana Kong, Mariana S Sousa, Janeane Harlum, Deborah Parker, Ravi Srinivas, Jennifer Wiltshire and Ajesh George in Palliative Medicine
