# Supplementary material for: The perceptions of palliative care medical practitioners towards oral health: A descriptive qualitative study
Source: Palliat Med. 2024 Mar 20;38(3):310–9. doi: 10.1177/02692163241233974 (PMC10958743; doi:10.1177/02692163241233974)
Supplement: sj-pdf-1-pmj-10.1177_02692163241233974 – Supplemental material for The perceptions of palliative care medical practitioners towards oral health: A descriptive qualitative study [file sj-pdf-1-pmj-10.1177_02692163241233974.pdf]

Supplemental material

<u>Topic guide for general practitioners (staff specialists) focus group</u>

What do you think about the importance of maintaining oral health for palliative care patients - why/why not?

What are the roles of staff specialists, in maintaining the oral health of patients: current practice?

What do you perceive would be the facilitators and barriers in implementing the targeted oral health program (risk assessment, prevention and referral) in the palliative care setting?

What are your recommendations on overcoming difficulties and barriers of delivering targeted oral health program in the palliative care setting?

Do you feel that staff specialists have the knowledge and confidence to provide oral health education, assessment and referrals to patients?

What specific further education and training skills are needed for staff specialists to provide oral health care program and referral services?

What would be the preferred content, duration and medium of training and education?

What are staff specialists' perceptions on using teledentistry in palliative care setting?

What do staff specialists perceive would be the facilitators and barriers on using teledentistry in the palliative care setting?
